# Investigating Overlapping Genetic Factors and Novel Causal Genes in Autoimmune Diseases: A Transcriptome-Wide Association and Multiomics Study

**DOI:** 10.1155/ijog/9595651

**Published:** 2025-06-24

**Authors:** Leihua Fu, Jieni Yu, Xin Wang, Zhe Chen, Jiaying Sun, Feidan Gao, Zhijian Zhang, Jiaping Fu, Pan Hong, Weiying Feng

**Affiliations:** ^1^Department of Hematology, Shaoxing People's Hospital, Shaoxing City, Zhejiang Province, China; ^2^Department of Genome Science and Microbiology, Faculty of Medical Sciences, University of Fukui, Fukui, Japan; ^3^Department of Rheumatology, Shaoxing People's Hospital, Shaoxing City, Zhejiang Province, China

**Keywords:** autoimmune disease, flotillin-1 (FLOT1), platelet, SLE, summary data–based Mendelian randomization (SMR)

## Abstract

**Background:** Autoimmune diseases exhibit familial clustering and co-occurrence, suggesting the presence of shared genetic risk factors. However, the overlapping genetic factors across these diseases have yet to be fully elucidated. This study aimed to identify shared genetic factors across five autoimmune diseases: systemic lupus erythematosus (SLE), rheumatoid arthritis (RA), ankylosing spondylitis (AS), Sjögren's syndrome (SS), and polymyalgia rheumatica (PMR).

**Methods:** A blood tissue–based transcriptome-wide association study (TWAS) was conducted to identify candidate genes. Bayesian colocalization analysis was employed to pinpoint genetic variants shared across diseases. Multiomics summary data–based Mendelian randomization (SMR) was used to identify causal risk genes, while transcriptomic analysis, gene set variation analysis (GSVA), and weighted gene coexpression network analysis (WGCNA) were applied to further investigate the functional roles of these genes.

**Results:** The TWAS identified 78 candidate genes across the five autoimmune diseases. Bayesian colocalization analysis revealed five genes, GTF2H4, FLOT1, HCP5, IER3, and STK19, that share genetic variants across these disorders. Specifically, RA and AS shared independent variants of GTF2H4 (rs2230365 and rs147708689, respectively). HCP5 variants were shared with SS (rs1800628) and SLE (rs1150757), and rs1800628 was also identified as a shared locus in FLOT1 for SLE. SMR analysis highlighted FLOT1 as a strong causal risk gene for SLE. Transcriptomic analysis showed that FLOT1 is highly expressed in T cells and platelets, with involvement in multiple metabolic pathways. WGCNA identified four key neighboring genes, EHD1, SLC10A3, LMNA, and STXBP2, associated with FLOT1.

**Conclusion:** This study uncovers shared genetic factors across five autoimmune diseases, with FLOT1 identified as a novel causal risk gene for SLE. These findings suggest that platelet-mediated pathogenic mechanisms may contribute to SLE, providing a potential target for future therapeutic interventions.

## 1. Introduction

Autoimmune diseases consist of a series of chronic inflammatory and destructive diseases, including systemic lupus erythematosus (SLE), rheumatoid arthritis (RA), and Sjogren's syndrome (SS) [1]. The familial aggregation, co-occurrence in epidemiological studies, and the cross-disease efficacy of therapeutic interventions highlight the intricate interconnections among these disorders [2]. Genome-wide association studies (GWAS), designed to identify common single nucleotide polymorphisms (SNPs) with modest to large effects on phenotype, have identified numerous loci harboring risk variants in autoimmune diseases [1, 3]. High rates of risk alleles are found to be shared between these diseases, raising the possibility of a molecular basis for their shared pathogenesis [4]. However, the majority (> 90%) of putative causal variants linked to autoimmune diseases are located in noncoding regions of the genome [2]. Consequently, the gene associations within autoimmune diseases remain largely unidentified at most GWAS loci.

More recently, transcriptome-wide association study (TWAS), which leverages expression quantitative trait loci (eQTL) reference panels to identify gene–trait associations from GWAS datasets, has successfully bridged the gap between disease-associated GWAS loci and specific genes, offering valuable insights into gene–trait relationships [5]. Several studies have successfully identified disease-related genes through TWAS, including those associated with SLE [6], RA [7], and ankylosing spondylitis (AS) [8]. However, although TWAS identifies associations between gene expression and autoimmune conditions, the underlying genetic connections remain insufficiently explored. In this context, Bayesian colocalization analysis, which estimates the probability that gene expression and traits share a common causal variant [9], offers a powerful approach to uncover the shared genetic architecture underlying autoimmune diseases.

To further prioritize putative causal genes, summary data–based Mendelian randomization (SMR) provides an efficient approach by integrating GWAS summary statistics with cis-eQTL data to infer causal relationships between gene expression and complex traits [10]. When combined with Bayesian colocalization analysis, SMR enables more stringent filtering of spurious associations by ensuring that gene–trait correlations arise from shared causal variants rather than linkage disequilibrium (LD) [11, 12]. This integrative approach improves the specificity and robustness of causal gene identification.

In this study, we aimed to identify shared genetic factors across autoimmune diseases and uncover novel causal genes. A comprehensive TWAS analysis was conducted, which revealed disease-associated candidate genes (DACGs), including overlapping genes across multiple disorders. Subsequent Bayesian colocalization analysis identified shared genetic variants between these genes and the corresponding diseases. To further prioritize causal genes, SMR analysis was applied, confirming 12 genes with putative causal roles. Among them, we focused on flotillin-1 (FLOT1), a novel SLE risk gene supported by both colocalization and SMR evidence. FLOT1 encodes a scaffolding protein involved in lipid raft formation [13, 14]. Although it has not been previously linked to SLE, disruptions in lipid metabolism, particularly involving lipid rafts and associated enzymes, are known to influence immune responses and contribute to SLE pathogenesis [15, 16]. To gain further insight into the role of FLOT1 in SLE, we conducted a multiomics-based SMR analysis to investigate its potential molecular mechanisms and further analyzed its biological functions and key neighboring genes in the SLE transcriptome.

## 2. Materials and Methods

### 2.1. Study Design

**Figure 1:**
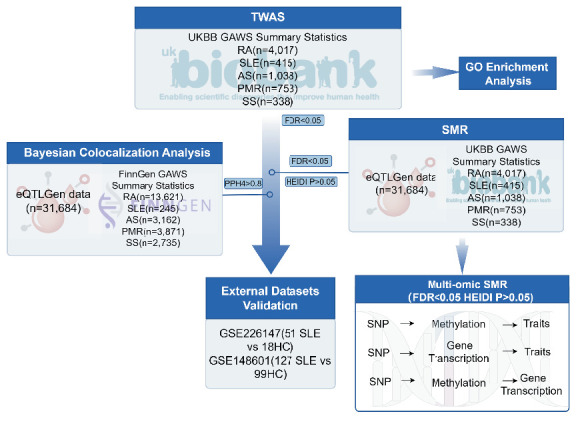
Workflow of the study. TWAS was first performed using GWAS summary statistics. Bayesian colocalization analysis was then conducted with GWAS and eQTL data to identify shared genetic variants (PPH4 > 0.8). SMR and multiomics SMR analyses were subsequently used to infer causal gene–disease relationships and explore underlying molecular mechanisms. Finally, external transcriptome datasets were used for validation, and network-based approaches were employed to characterize the biological functions of the identified causal genes.

The overview of this study is illustrated in Figure 1. Initially, we performed a TWAS for five autoimmune diseases, including RA, SLE, SS, AS, and polymyalgia rheumatica (PMR), to investigate DACGs. Subsequently, a Bayesian colocalization analysis was conducted to identify shared variants, followed by a multi-SNP SMR (SMR-multi) analysis to explore the causal relationships between DACGs and disorders. For the causal genes that passed these two analyses, a multiomics-based SMR was performed to identify potential molecular mechanisms. Finally, the expression of causal genes was validated in external transcriptome datasets, and potential gene functions were analyzed through gene set variation analysis (GSVA). Key neighboring genes were identified using weighted gene coexpression network analysis (WGCNA).

### 2.2. Data Resources

TWAS eQTL reference panel used for predicting gene expression was derived from the Young Finns Study (*YFS*, *n* = 1264) and was downloaded from the FUSION website [17]. GWAS summary statistics were obtained from the UK Biobank (UKBB) and FinnGen databases. Blood eQTL summary statistics were retrieved from eQTLGen Consortium, which encompasses genetic data on blood gene expression from 31,684 individuals across 37 datasets [18]. Blood methylation quantitative trait locus (mQTL) summary statistics came from a meta-analysis of two European cohorts: the Brisbane Systems Genetics Study (*n* = 614) and the Lothian Birth Cohorts (*n* = 1366), provided by Yang Lab [19]. Our study focused on *cis*-eQTL and *cis*-mQTL, defined as SNPs located within 1 and 2-Mb distances from the start and end of the gene, respectively.

Transcriptome datasets were downloaded from the Gene Expression Omnibus (GEO) database. The GSE148601 dataset contains mRNA expression profiles of major peripheral blood cell subsets from 127 SLE patients and 99 healthy controls. A total of 22.8% (29/127) of SLE patients were untreated at the time of sampling, and only these individuals were selected for downstream analysis. GSE226147 includes platelet transcriptomic data from 51 SLE patients and 18 controls. GSE173670 provides monocyte transcriptomes from 12 primary SS patients and 11 controls, with 58% (7/12) untreated. GSE117769 contains white blood cell expression data from 50 RA patients and 50 matched healthy controls; 14% (7/50) were treatment-naïve, and only these were included in the analysis. More detailed information about the datasets is provided in Table S1.

### 2.3. TWAS of Autoimmune Disease

We conducted a TWAS using the FUSION pipeline with default parameters, employing the *YFS* reference panel on UKBB GWAS summary statistics for five autoimmune diseases. The FUSION method constructs predictive models for the genetic components of functional or molecular phenotypes and estimates their association with diseases using GWAS summary statistics [17]. We applied a Benjamini–Hochberg correction to account for multiple testing, considering a false discovery rate (FDR) < 0.05 as a significant gene association [20], thereby identifying DACGs.

### 2.4. Functional Enrichment Analysis

For functional annotation of the DACGs from TWAS, gene ontology (GO) analysis was performed using the *org.Hs.eg.db R* package [21]. Significant GO terms and signaling pathways were identified with thresholds of adj.*p*.val < 0.05 (Benjamini–Hochberg correction) and *q* value < 0.05.

### 2.5. Bayesian Colocalization Analysis of Autoimmune Disease–Associated Genes

To further investigate whether autoimmune diseases share the same variants as DACGs, we performed Bayesian colocalization analysis [9]. Summary-level GWAS data for the disease were obtained from the FinnGen database, and the corresponding eQTLs for DACGs were extracted from the eQTLgen database, as described above, using SNPs within ±1-Mb gene regions as instruments. Analysis was performed using the *coloc* R package to obtain the posterior probabilities for five hypotheses (H0–H4) in a Bayesian framework. A posterior probability for H3 (PP.H3) > 0.8 indicates an association with gene expression and disease in an independent SNP, while PP.H4 > 0.8 indicates strong evidence for a shared variant affecting both gene expression and disease [9].

### 2.6. SMR

SMR-multi is an analysis to test if the effect size of SNPs on the phenotype is mediated by gene expression [19]. The SNPs responsible for molecular QTLs, including eQTL and mQTL, are pruned for LD using a weighted vertex coverage algorithm with an LD *r*^2^ threshold (default value = 0.9) and selected as instruments [19].

A multiomics SMR was performed as described below: (1) responsible blood eQTL SNPs were selected as instruments, DACG expressions were exposure, and autoimmune diseases were outcome; (2) responsible blood mQTL SNPs were selected as instruments, DNA methylations were exposure, and autoimmune diseases were outcome; and (3) responsible blood mQTL SNPs were selected as instruments, DNA methylations were exposure, and DACG expressions were outcome [22]. We set a threshold of genome-wide significance (*p* < 5 × 10^−8^) in all eQTL, mQTL, and GWAS. An FDR < 0.05 and heterogeneity in the dependent instrument (HEIDI) test results with *p* > 0.05 were considered significant in analysis [10].

### 2.7. Identification of Differentially Expressed Genes (DEGs) and GSVA

The expression of causal gene was initially analyzed statistically using the Wilcoxon test for pairwise comparisons in transcriptome datasets, with *p* < 0.05 set as the threshold for statistical significance. Then, the *limma* R package was used to screen for DEGs with the criteria of |log2 fold change (FC)| > 0.5 and adj.*p*.val < 0.05. Receiver operating characteristic (ROC) curve analysis was performed to evaluate the diagnostic value of causally associated genes for relevant autoimmune diseases using the *pROC* R package [23]. GSVA was performed to annotate the differential status of metabolic pathways between patients and healthy controls using the *GSVA* R package with hallmark gene sets (h.all.v2024.1.Hs.symbols). Pathways with adj.*p*.val < 0.05 and |log2FC| > 0.15 were considered statistically significant [24].

### 2.8. WGCNA

WGCNA was conducted to identify gene modules with strong correlations, examine the relationships between modules, and assess their connections to clinical phenotypes [25]. In our study, we used the *WGCNA* R package to identify the modules most closely associated with disease. The optimal soft threshold was determined to convert the correlation matrix into an adjacency matrix, followed by the construction of a topological overlap matrix (TOM) from the adjacency matrix. The module exhibiting the strongest positive correlation with disease was selected for further investigation through the calculation of the Pearson correlation coefficient between the modules and the disease. Gene–trait significance (GS) and module membership (MM) were computed for the most correlated module, with hub genes selected based on MM > 0.75 and GS > 0.2 [26]. Finally, the hub genes' network was constructed by Cytoscape software (v.3.10.3).

### 2.9. Statistical Analysis

All statistical analyses were performed using *R* software (v.4.3.2). The Wilcoxon test was used for pairwise comparisons, with *p* < 0.05 considered statistically significant. Pearson correlation analysis was conducted to assess the correlation between genes and pathways, with *p* < 0.05 considered statistically significant.

## 3. Results

### 3.1. TWAS of Autoimmune Disease and DACG Functional Enrichment Analysis

In a whole blood tissue–based TWAS of autoimmune diseases, a total of 78 DACGs showed a statistically significant signal after Benjamini–Hochberg correction (FDR < 0.05), including 6 genes in AS, 36 genes in RA, 15 genes in SLE, 9 genes in SS, and 12 genes in PMR (Table S2).

**Figure 2:**
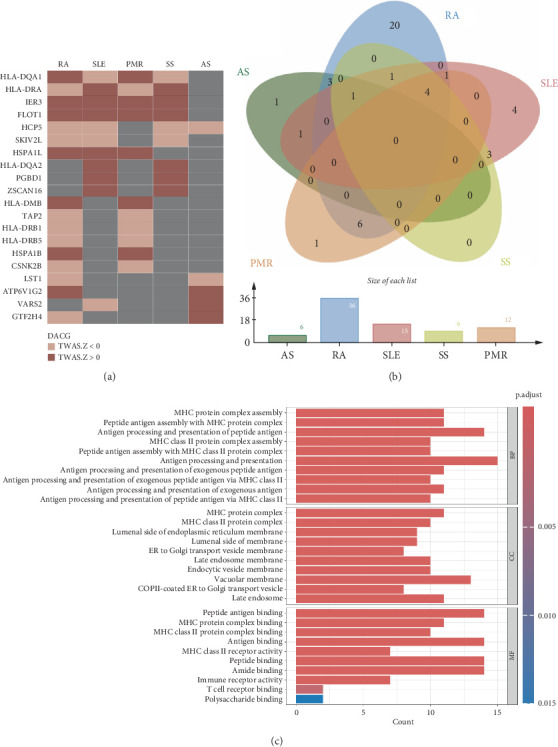
TWAS and functional annotation of DACGs in autoimmune diseases. (a) Heatmap showing overlapping DACGs identified by TWAS across five autoimmune diseases: RA, SLE, AS, PMR, and SS. TWAS *Z* score < 0 indicates a negative association between gene expression and disease risk, whereas a *Z* score > 0 indicates a positive association. (b) Venn diagram illustrating the overlap of DACGs across the five autoimmune diseases. The bar plot below displays the total number of DACGs identified in each disease. (c) GO enrichment analysis of the identified DACGs. Enriched terms are classified into biological processes (BPs), cellular components (CCs), and molecular functions (MFs). The bar length indicates the number of genes associated with each term, and the color represents the adjusted *p* value, with deeper shades indicating greater significance.

We found multiple overlapping genes across these diseases (Figure 2a,b). For instance, four genes, general transcription factor IIH subunit 4 (GTF2H4), human cytomegalovirus promoter 5 (HCP5), ATPase H+ transporting V1 subunit G2 (ATP6V1G2), and leukocyte specific transcript 1 (LST1), were detected in both RA and AS. The GTF2H4 gene was recognized as a positive-related gene in AS (TWAS.Z = 4.0332, FDR = 0.0423), while it was identified as a negative-related gene in RA (TWAS.Z = −6.6714, FDR = 11.8 × 10^−8^). Similarly, the FLOT1 gene was identified in RA (TWAS.Z = 6.9646, FDR = 1.71 × 10^−9^), SLE (TWAS.Z = 6.3121, FDR = 1.16 × 10^−6^), SS (TWAS.Z = 5.3636, FDR = 1.79 × 10^−4^), and PMR (TWAS.Z = 3.9294, FDR = 3.32 × 10^−2^) and was recognized as a positive-related gene in all of these diseases. Additionally, we identified 9 genes overlapping between SLE and SS, 5 genes overlapping between SLE and PMR, and 11 genes overlapping between RA and PMR.

A GO analysis revealed that these DACGs are involved in the assembly of major histocompatibility complex (MHC) class II protein complexes and in peptide antigen binding. Notably, we identified critical restriction elements essential for CD4+ T cell proliferation and differentiation, including human leukocyte antigen DRA (HLA-DRA), HLA-DRB1, and HLA-DMB (Figure 2c).

### 3.2. Bayesian Colocalization Analysis Identifies Shared SNPs Between DACGs and Autoimmune Disease

**Figure 3:**
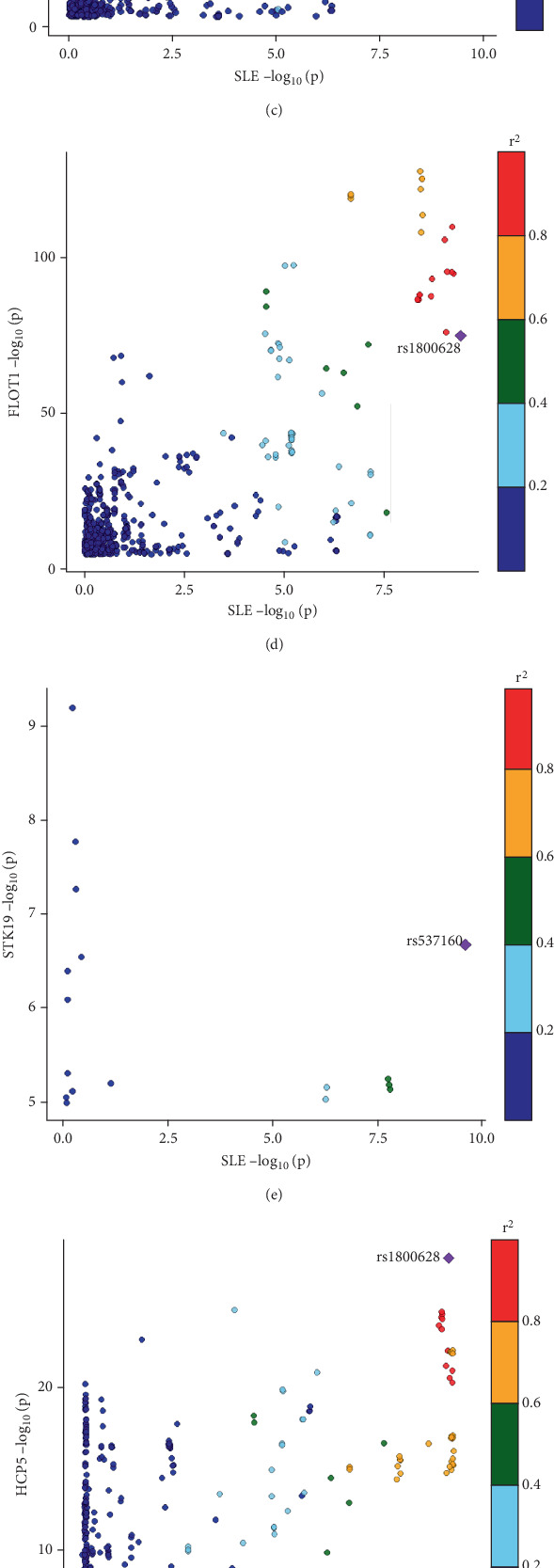
Bayesian colocalization analysis of DACG eQTLs and autoimmune disease GWAS signals. (a) Dot plot showing the colocalization posterior probabilities between DACG eQTL signals and GWAS loci from five autoimmune diseases. The size and color of the dots represent the posterior probabilities for shared genetic signals: PP.H4 (shared causal variant between eQTL and GWAS signal) and PP.H3 (distinct causal variants). Larger and darker dots indicate higher posterior probabilities. (b–h) Regional plots showing colocalization between DACG eQTLs and GWAS signals for specific gene–disease pairs with strong evidence of a shared causal variant. Each dot represents a genetic variant; the color scale denotes LD (*r*^2^) with the lead SNP.

Our Bayesian colocalization analysis revealed five genes, including GTF2H4, FLOT1, HCP5, immediate early response 3 (IER3), and serine/threonine kinase 19 (STK19), that were identified as having a shared variant with autoimmune diseases (PP.H4 > 0.8, Figure 3a and Table S2).

Specifically, our analysis identified an overlapping gene between AS and RA, GTF2H4, which harbored two independent variants, rs147708689 and rs2230365, shared by AS and RA, respectively (Figure 3g,h). Similarly, another overlapping gene, HCP5, harbored distinct variants rs1800628 and rs1150757, which were shared by SS and SLE, respectively (Figure 3b,f). Notably, we identified a pleiotropic functional variant, rs1800628, as a shared variant associated not only with the FLOT1 gene in SLE but also with the HCP5 gene in SS (Figure 3d,f).

### 3.3. Identification of Causal Genes in Autoimmune Diseases and Exploration of Potential Molecular Mechanisms

To further explore potential causal relationships between these DACGs and autoimmune diseases, we performed SMR-multi analysis and successfully identified 12 causal gene–disease pairs (Table 1 and Table S3). Among them, we highlighted a novel causal gene, FLOT1, which harbors a shared variant (rs1800628) with SLE (PP.H4 = 0.95, Figure 3d and Table 1), suggesting that it may play a pivotal role in the pathogenesis of SLE. Notably, FLOT1 was the only gene found to both share a genetic variant and be identified as a causal gene across the five autoimmune disorders. Therefore, we further investigated the molecular mechanisms and biological functions of FLOT1 in the context of SLE.

**Figure 4:**
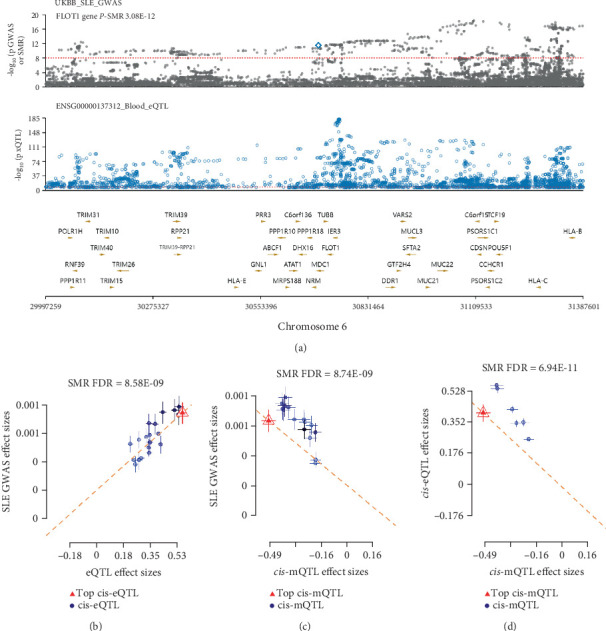
Multiomics SMR analysis reveals causal associations between FLOT1 and SLE. (a) LocusZoom plot showing the genomic position of the FLOT1 gene on Chromosome 6 based on UK Biobank SLE GWAS summary statistics (top panel) and blood eQTL data from the eQTLGen consortium (bottom panel). (b–d) Multiomics SMR analyses evaluating the causal relationships among FLOT1 expression, DNA methylation, and SLE susceptibility. (b) SMR analysis between FLOT1 expression and SLE GWAS data demonstrates a significant positive association (FDR = 8.58 × 10^−9^), supporting FLOT1 as a potential risk gene for SLE. (c) SMR analysis between FLOT1 methylation and SLE GWAS reveals a significant inverse association (FDR = 8.74 × 10^−9^), suggesting that FLOT1 DNA methylation may serve as a protective factor against SLE. (d) SMR analysis between FLOT1 methylation and gene expression (FDR = 6.94 × 10^−11^) indicates a strong negative correlation, implying that increased DNA methylation is causally negatively associated with FLOT1 expression.

To uncover the potential molecular mechanisms of FLOT1 in SLE, a multiomics-based SMR analysis was performed. By incorporating blood DNA methylation and SLE GWAS data, we identified a total of 967 causal mQTL DNA methylation sites (Table S4), including five sites within the FLOT1 gene. This finding led us to hypothesize that FLOT1 gene expression is regulated by DNA methylation, which in turn affects the risk of SLE. Subsequently, 12 DNA methylation sites were identified as having a causal association with the FLOT1 gene (Table S5). Among them, we identified a methylation site located 2 kb upstream of the CpG island, which not only showed a causal association with FLOT1 (Probe ID cg23423329, beta__SMR_ = −0.3607, *p*__SMR_ = 2.38 × 10^−11^, FDR = 6.94 × 10^−11^, *p*_⁣_HEIDI_ = 0.81; Figure 4d) but also acted as a protective factor against SLE (beta__SMR_ = −0.00024, *p*__SMR_multi_ = 4.04 × 10^−10^, FDR = 8.74 × 10^−9^, *p*_⁣_HEIDI_ = 0.51; Figure 4c). Taken together, we propose a potential molecular mechanism in which DNA methylation 2 kb upstream of the CpG island of FLOT1 downregulates gene expression (beta__SMR_ = −0.3607), thereby decreasing the risk of SLE (beta__SMR_ = −0.00024).

### 3.4. Elevated FLOT1 Expression in SLE and Identification of Key Metabolic Pathway Associations

**Figure 5:**
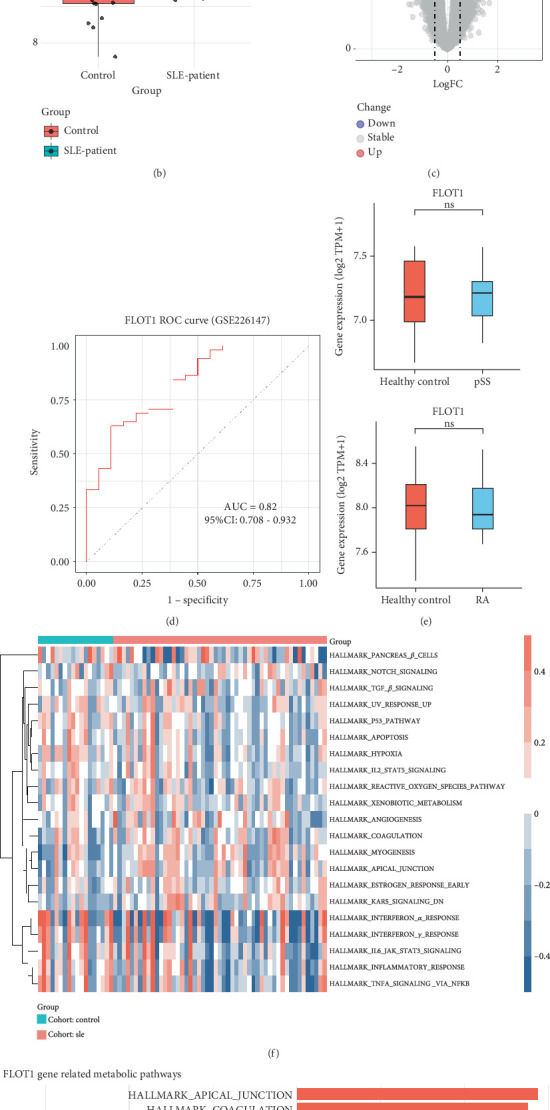
Expression pattern and functional annotation of FLOT1 in SLE. (a) FLOT1 expression across major immune cell subsets in PBMCs from SLE patients and healthy controls (GSE148601). Significantly elevated expression was observed in T cells from SLE patients. (b) FLOT1 expression in platelets from SLE patients versus healthy controls (GSE226147), showing marked upregulation in the SLE group. (c) Volcano plot of DEGs in SLE platelets (GSE226147), with FLOT1 highlighted among the significantly upregulated genes. (d) ROC curve demonstrating the diagnostic potential of FLOT1 expression in distinguishing SLE patients from healthy controls. (e) Box plots showing FLOT1 expression in primary SS versus healthy controls (top, GSE173670) and in RA versus healthy controls (bottom, GSE117769). No significant difference in FLOT1 expression was observed in either pSS or RA compared to healthy controls. (f) Heatmap displaying significantly enriched metabolic pathways in SLE platelets (GSE226147). (g) Pearson correlation analysis between FLOT1 expression and hallmark metabolic pathways.

Given that FLOT1 has been implicated in immune cell activation, we initially analyzed its expression across peripheral immune cell subsets using transcriptomic data from untreated SLE patients. We found that FLOT1 expression was selectively elevated in T cells (Figure 5, *p* = 0.0339), whereas no significant differences were observed in other cell types, including B cells, monocytes, and myeloid dendritic cells (mDCs). Since FLOT1 was also implicated as a candidate gene in other autoimmune diseases, including RA and SS, we subsequently assessed its expression in these cohorts. However, no significant differences in FLOT1 expression were observed between patients and healthy controls (Figure 5).

Recently, increasing evidence has highlighted the pivotal role of platelets in initiating and amplifying inflammatory responses, implicating them as active participants in the pathogenesis of SLE [27–29]. Moreover, lipid rafts, which are membrane microdomains formed with scaffolding proteins such as FLOT1, have been found to be involved in platelet activation [30]. These findings prompted us to investigate the potential involvement of FLOT1 in platelet activation in SLE. As expected, FLOT1 was significantly upregulated in SLE platelets and identified as one of the DEGs (Figure 5, *p* = 6.1 × 10^−5^, Table S6). Further ROC analysis indicated its substantial diagnostic potential (Figure 5, AUC = 0.82, 95% CI 0.708–0.932). GSVA revealed 21 differentially enriched metabolic pathways in platelets from SLE patients (Figure 5 and Table S7). FLOT1 expression was strongly correlated with the APICAL_JUNCTION (*r*__Pearson_ = 0.72, *p* = 3.28 × 10^−12^), COAGULATION (*r*__Pearson_ = 0.69, *p* = 5.08 × 10^−11^), REACTIVE_OXYGEN_SPECIES_PATHWAY (*r*__Pearson_ = 0.58, *p* = 2.08 × 10^−7^), MYOGENESIS (*r*__Pearson_ = 0.68, *p* = 1.17 × 10^−10^), and UV_RESPONSE_UP (*r*__Pearson_ = 0.58, *p* = 1.38 × 10^−7^) pathways (Figure 5 and Table S8).

### 3.5. WGCNA Reveals FLOT1 as a Key Hub Gene in SLE Platelets With Significant Neighboring Gene Associations

**Figure 6:**
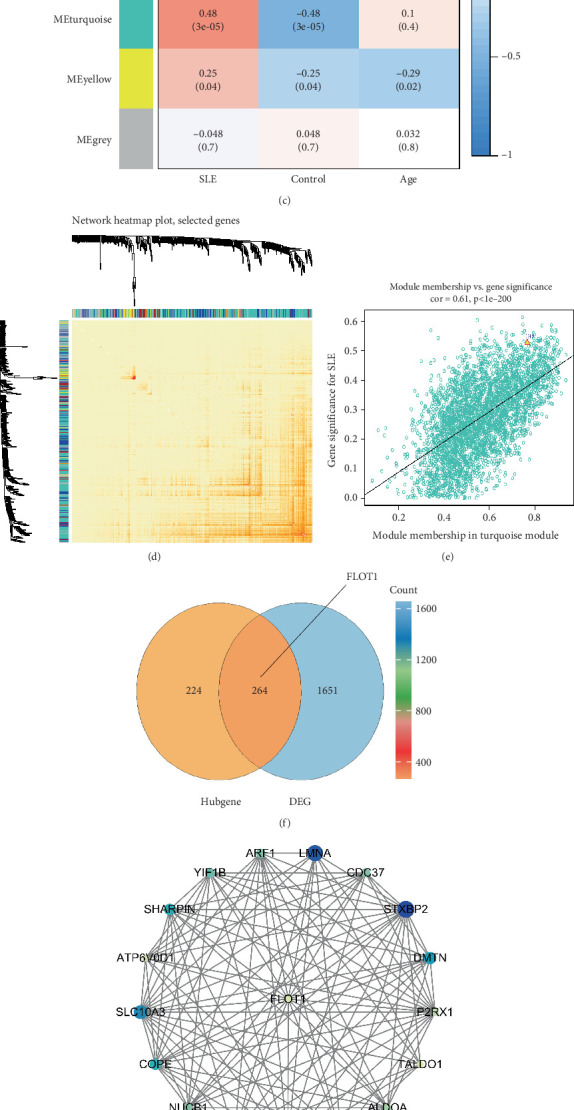
WGCNA and FLOT1-centered gene network analysis in SLE platelets. (a–e) WGCNA performed on the GSE226147 dataset (SLE platelet transcriptome). (a) Gene clustering dendrogram and identification of gene coexpression modules, each represented by a unique color. (b) Analysis of scale-free topology model fit to determine the optimal soft-thresholding power. (c) Module–trait correlation heatmap showing Pearson correlations between each module eigengene and SLE. The turquoise module exhibited the strongest positive correlation with SLE. (d) Heatmap of gene connectivity within modules, highlighting the turquoise module. (e) Scatterplot showing the relationship between module membership and gene significance for genes in the turquoise module, indicating a strong positive correlation (cor = 0.61, *p* < 2 × 10^−200^). (f) Venn diagram showing the overlap between DEGs and hub genes from the turquoise module; FLOT1 is included in the intersecting set. (g) Gene network centered on FLOT1, showing its top coexpressed neighboring genes within the turquoise module. (h) Pearson correlation analysis between FLOT1 and representative neighboring genes, indicating strong coexpression relationships.

To further investigate FLOT1-associated neighboring genes in SLE platelets, we performed WGCNA. By constructing gene modules and selecting the optimal soft threshold (Figure 6), we identified the turquoise module as having the highest correlation with SLE (Figures 6, 6, and 6), ultimately identifying 488 hub genes within this module (Table S9). A Venn diagram of DEGs and hub genes revealed FLOT1 as an intersecting gene (Figure 6). Next, we calculated the degree values of hub genes using Cytoscape, identifying 17 neighboring genes (Figure 6). Pearson correlation analysis further demonstrated that FLOT1 expression was significantly correlated with EH domain-containing 1 (EHD1), solute carrier family 10 member 3 (SLC10A3), lamin A/C (LMNA), and syntaxin binding protein 2 (STXBP2) (Figure 6).

## 4. Discussion

Approximately two-thirds of the variants associated with 21 autoimmune diseases have been reported to reside in overlapping loci [2]. However, as the majority of these loci are located in noncoding regions, elucidating the biological functions of the associated variants remains a major challenge. In this study, we explored the shared genetic factors of autoimmune diseases using a more integrative and explanatory approach. Initially, we performed a TWAS analysis, a powerful approach for linking genetically regulated gene expression to complex traits, and identified 78 genes associated with five autoimmune diseases. We then performed Bayesian colocalization analysis to investigate whether the identified genes and autoimmune diseases share common genetic variants. As a result, we identified five genes with strong evidence of shared genetic loci associated with autoimmune diseases. By comparing these locus–disease relationships, we identified two distinct patterns of genetic sharing.

In the first pattern, a single gene contains two distinct genetic variants, each associated with a different disease. For example, within the HCP5 gene, the variants rs1800628 and rs1150757 were colocalized with SS and SLE, respectively (Figure 3b,f). The genetic association between the HCP5 gene and SLE has been previously reported in a cohort of Italian patients. In that study, the association was driven by a different variant, rs3099844 (*p* = 0.01, OR = 2.06, 95% CI: 1.18–3.6) [31]. The discrepancy in lead variants may be partially explained by population-specific genetic diversity [32]. Another example of this pattern is the GTF2H gene, which contains two distinct variants: rs2230365 (PP.H4 = 0.8858), colocalized with RA, and rs147708689 (PP.H4 = 0.8887), colocalized with AS. Although the genetic variants differ between diseases and thus do not constitute true genetic sharing in the strict sense, they nevertheless implicate the same gene in the pathogenesis of both conditions.

The second pattern represents a scenario in which a single genetic variant influences multiple diseases. For instance, rs1800628 was identified as a shared variant both in SLE and SS, suggesting the presence of shared molecular mechanisms underlying both conditions [2]. This observation is consistent with the substantial clinical overlap between SLE and SS. Importantly, identifying such shared variants may facilitate the expansion of therapeutic applications across diseases. A notable example is the low-frequency missense variant rs34536443 in the tyrosine kinase 2 (TYK2) gene, which encodes a nonreceptor tyrosine kinase broadly expressed in immune cells. This variant confers strong protection against 10 autoimmune diseases [33]. These findings suggest that pharmacological inhibition of TYK2 may be effective across a broad spectrum of autoimmune disorders. In support of this, the first selective TYK2 inhibitor, deucravacitinib, is for the treatment of plaque psoriasis, with ongoing clinical trials in psoriatic arthritis, SLE, Crohn's disease, and ulcerative colitis. TYK2 inhibitors may thus hold promise for additional autoimmune indications [33].

Although TWAS identified candidate genes associated with autoimmune diseases, it remains unclear which of these have causal roles. To address this, we performed SMR-multi analysis and identified 12 genes with evidence of causal associations (Table 1). Among these, FLOT1 emerged as a novel risk gene for SLE, as it was the only causal gene found to harbor a shared variant with the disease (rs1800628; Figure 3d; PP.H4 = 0.95). Moreover, we identified a DNA methylation site (Probe ID cg23423329) located 2 kb upstream of the CpG island that showed a significant negative causal association with FLOT1 expression (Figure 4d). As FLOT1 was also identified as a DACG in the TWAS analysis, we further examined its expression in RA and SS. However, no significant differences in FLOT1 expression were observed between patients and healthy controls in either disease (Figure 5e). Therefore, we focused on investigating the molecular mechanisms and biological functions of the FLOT1 gene in SLE.

FLOT1, located at 6p21.33, encodes a membrane-associated scaffolding protein essential for the formation of lipid rafts, which are critical for signal transduction [13, 14]. Studies suggest that FLOT1 contributes to immune cell polarity and T cell migration by stabilizing lipid raft domains and organizing signaling complexes at the immunological synapse [34]. Notably, altered lipid raft composition has been observed in SLE T cells, where increased cholesterol and gangliophospholipid levels promote aberrant accumulation of signaling molecules such as CD45 and Lck, thereby lowering activation thresholds and sustaining TCR signaling [35, 36]. Intriguingly, a similar pathogenic mechanism has been reported for Fc*γ*RIIb, an inhibitory receptor expressed on B cells and myeloid cells. A common SLE-associated polymorphism (Fc*γ*RIIbT232), which substitutes a threonine in the transmembrane domain, disrupts raft targeting and leads to exclusion of the receptor from lipid rafts. As a result, Fc*γ*RIIbT232 fails to suppress activating Fc*γ*R signaling, contributing to enhanced antigen presentation and cytokine production [37, 38]. These studies collectively suggest that dysregulation of the spatial architecture of lipid raft-based signaling hubs may facilitate persistent immune activation in SLE, in which FLOT1, as a core structural component of lipid rafts, may play a contributory role. Our further analysis of transcriptomic data from SLE patient datasets demonstrated elevated expression of FLOT1 in T cells, indicating its potential involvement in the activation or dysregulation of immune responses in SLE (Figure 5a). Future studies are warranted to elucidate the precise molecular mechanisms by which FLOT1 contributes to T cell dysfunction and immune imbalance in SLE.

**Figure 7:**
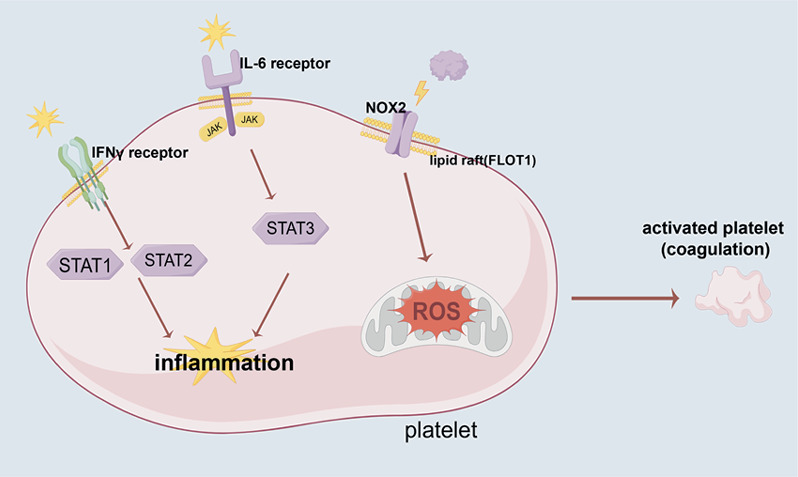
FLOT1 integrates interferon, ROS, and coagulation signaling pathways in SLE platelets. This schematic illustrates the central role of FLOT1, a lipid raft–associated scaffolding protein, in coordinating key signaling events in SLE platelets. Transcriptomic analysis revealed significant upregulation of FLOT1 in platelets from SLE patients, with positive correlations to interferon-related, IL6/STAT3, ROS, and coagulation pathways. NOX2: NADPH oxidase 2. STAT: signal transducer and activator of transcription.

Increasing evidence has implicated platelets as active participants in the pathogenesis of SLE [27–29] exhibiting an activated phenotype characterized by elevated surface expression of P-selectin, increased granule release, and enhanced production of proinflammatory extracellular vesicles [29]. Upon activation, platelets secrete soluble mediators such as CD40 ligand, interleukin-1*β* (IL-1*β*), and S100A8/A9 [28]. These molecules stimulate endothelial cells, plasmacytoid dendritic cells, and monocytes, thereby promoting proinflammatory cytokine release [28]. Recent studies indicate that lipid rafts serve as critical membrane platforms facilitating the spatial organization of signaling molecules during platelet activation, thereby enhancing receptor clustering and downstream signal propagation [39–41], although the specific involvement of FLOT1 in this process remains unclear. In our transcriptomic analysis of platelets from SLE patients, we observed significant activation of immune–inflammatory pathways, including interferon response–related pathways, inflammatory response pathways, and IL6/STAT3 signaling (Figure 5f). Notably, FLOT1 was significantly upregulated in SLE platelets (Figure 5b) and was positively correlated with these immune–inflammatory pathways (Figure 5g and Table S8), suggesting that FLOT1 may be involved in platelet-mediated immune responses and signal transduction, thereby contributing to the pathogenesis of SLE (Figure 7).

In addition to its involvement in immune–inflammatory processes, we found that FLOT1 is strongly associated with signaling pathways related to reactive oxygen species (ROS) and coagulation (Figure 5g, Table S8). ROS play a pivotal role in modulating platelet activation and thrombosis. Specifically, NADPH oxidase 1 (NOX1)- and NOX2-derived ROS promote platelet aggregation through the Syk–PLC*γ*–Ca^2+^ signaling pathway, with NOX2 being essential for arterial thrombosis [42]. Moreover, mitochondrial ROS contribute to platelet hyperactivation under pathological conditions by inducing mitochondrial permeability transition pore formation and apoptosis, ultimately leading to thrombus formation [43] (Figure 7). Given that SLE is associated with a markedly elevated risk of cardiovascular morbidity [44] and thrombotic events [45], the prominent involvement of FLOT1 in platelet coagulation pathways underscores the importance of elucidating its role in SLE-related cardiovascular disease and thrombosis.

We further identified hub genes associated with SLE platelets through WGCNA and constructed a FLOT1-centered network (Figure 6g), which revealed four key neighboring genes: EHD1, SLC10A3, LMNA, and STXBP2 (Figure 6h). Some of these genes have been reported as pivotal in SLE. For example, LMNA proteins, encoded by the LMNA gene, are linked to SLE pathogenesis; autoantibodies against lamin C are associated with systemic secondary vasculitis in SLE [46], and LMNA mRNA expression has been identified as a specific antiviral response to type I interferon (IFN) activation in chilblain lupus erythematosus [47]. Another gene, STXBP2, has also been implicated in SLE-related macrophage activation syndrome [48]. The strong association of the FLOT1 gene with these pivotal neighboring genes provides critical insights into the role of platelets in SLE pathogenesis.

While our findings are promising, several limitations merit discussion. First, our TWAS methodology, which exclusively evaluates the *cis*-genetic component of gene expression, is inherently limited in its ability to capture *trans*-eQTL effects. Future research should prioritize the establishment of more extensive gene expression reference panels to enable a comprehensive investigation of *trans*-eQTL effects. Second, the potential confounding effects of immunosuppressive therapy are a concern in transcriptomic studies of autoimmune diseases. In the platelet transcriptomic dataset, SLE patients were receiving immunosuppressive agents. Although our pathway analysis indicated that immune-related pathways remained activated, suggesting minimal interference from medication, the study was not adequately powered to incorporate medication use into the analysis. Third, autoimmune diseases are not tissue-restricted conditions. Our study focused on blood tissue, the most accessible and commonly used sample type; a multitissue transcriptomic analysis involving other tissues such as joints, kidneys, and skin could yield more comprehensive and insightful conclusions.

In summary, our study utilized TWAS to identify candidate and overlapping genes in five autoimmune diseases. Bayesian colocalization analysis revealed shared genetic influences across these diseases, suggesting common pathways. We identified FLOT1 as a causal risk gene for SLE and further explored its biological function in SLE platelets to better understand its potential mechanism. These findings enhance our understanding of autoimmune disease genetics and could inform future research and therapeutic strategies.

## Figures and Tables

**Table 1:** FLOT1 identified as a robust causal gene through integrative SMR and colocalization analysis. All genes listed showed significant associations with disease risk based on SMR analysis (FDR < 0.05), indicating potential causal roles. Among them, FLOT1 was uniquely supported by both SMR and Bayesian colocalization analysis, with a high posterior probability of a shared causal variant (PP.H4 = 0.95), suggesting strong evidence for a shared genetic basis between FLOT1 expression and SLE susceptibility.

**Traits**	**Gene**	**SMR**				**COLOC**	
		**B** **e** **t** **a**__**SMR**_	**p**__**SMR**_**multi**_	**FDR**	**Causal gene**	**PP.H4**	**Share variant**
AS	CD164	0.001	9.06 × 10^−5^	1.09 × 10^−4^	Yes	2.21 × 10^−3^	No

PMR	HSPA1L	0.0011	5.30 × 10^−8^	9.09 × 10^−8^	Yes	2.50 × 10^−19^	No
	HLA-DMB	0.0013	0.02	0.03	Yes	3.27 × 10^−5^	No

RA	MAPK3	−0.0017	0.03	0.03	Yes	0.07	No

SLE	BTN3A2	−0.0003	5.22 × 10^−4^	7.83 × 10^−4^	Yes	0.02	No
	FLOT1	0.0013	3.43 × 10^−9^	8.58 × 10^−9^	Yes	0.95	Yes
	VARS2	−0.0008	3.76 × 10^−8^	7.04 × 10^−8^	Yes	0.01	No
	SKIV2L	−0.0004	2.78 × 10^−13^	4.17 × 10^−12^	Yes	7.15 × 10^−8^	No
	HLA-DRA	0.0011	1.29 × 10^−5^	2.15 × 10^−5^	Yes	2.28 × 10^−5^	No

SS	ZSCAN16	0.0028	2.30 × 10^−5^	2.96 × 10^−5^	Yes	6.87 × 10^−10^	No
	SKIV2L	−0.0003	4.83 × 10^−13^	1.09 × 10^−12^	Yes	4.18 × 10^−75^	No
	HLA-DRA	0.0005	6.15 × 10^−5^	6.91 × 10^−5^	Yes	2.00 × 10^−10^	No

## Data Availability

This study utilized only publicly available data, with detailed information on data sources and handling provided in the Materials and Methods section and Supporting Information tables. The TWAS *cis*-eQTL reference panel, used for predicting gene expression, was obtained from the *YFS* and downloaded from the FUSION website (http://gusevlab.org/projects/fusion). GWAS summary statistics for five rheumatoid clinical subtypes were sourced from the UK Biobank (https://www.ukbiobank.ac.uk) and FinnGen (https://www.finngen.fi/en) databases. Blood eQTL summary statistics were retrieved from the eQTLGen Consortium (https://www.eqtlgen.org). Blood mQTL summary statistics came from a meta-analysis of two European cohorts: the Brisbane Systems Genetics Study and the Lothian Birth Cohorts, provided by Yang Lab (https://yanglab.westlake.edu.cn). GEO datasets were downloaded from NCBI (https://www.ncbi.nlm.nih.gov/geo/).

## Supporting Information

Supporting Information 1 Table S1: Datasets used in this study.

Supporting Information 2 Table S2: Transcriptome-wide association studies (TWAS) and colocalization analysis of autoimmune diseases (FDR < 0.05).

Supporting Information 3 Table S3: Summary data–based Mendelian randomization (SMR) analysis from blood gene expression to autoimmune diseases (FDR < 0.05).

Supporting Information 4 Table S4: SMR analysis from blood DNA methylation to SLE (FDR < 0.05).

Supporting Information 5 Table S5: SMR analysis from blood DNA methylation to FLOT1 expression (FDR < 0.05).

Supporting Information 6 Table S6: Differentially expressed genes (DEGs) of platelet transcriptome in patients with SLE (GSE226147, |logFC| > 0.5 and adj.*p*.val > 0.05).

Supporting Information 7 Table S7: Significant metabolic pathways between SLE patients and healthy controls (GSE226147, adj.*p*.val < 0.05 and |logFC| > 0.15).

Supporting Information 8 Table S8: Metabolic pathways significantly correlated with FLOT1 gene expression in SLE patients (*p* < 0.05).

Supporting Information 9 Table S9. Hub genes in the turquoise module identified by WGCNA (GSE226147, geneModuleMembership > 0.75 and geneTraitSignificance > 0.2).
